# Oral Manifestations: A Warning-Sign in Children with Hematological Disease Acute Lymphocytic Leukemia

**DOI:** 10.3390/hematolrep15030051

**Published:** 2023-08-24

**Authors:** Sandra Clara Soares, Louis J. D. Roux, Ana Rita Castro, Cristina Cardoso Silva, Rita Rodrigues, Viviana M. P. Macho, Fátima Silva, Céu Costa

**Affiliations:** 1 Faculdade de Ciências da Saúde, Universidade Fernando Pessoa, 4200-150 Porto, Portugal; 2 Instituto de Investigação, Inovação e Desenvolvimento Fernando Pessoa, FP-I3ID (FP-BHS), 4249-004 Porto, Portugal; fsilva@ufp.edu.pt (F.S.);; 3 Escola Superior da Saúde, Universidade Fernando Pessoa, 4200-253 Porto, Portugal; 4 Grupo de Patologia Experimental e Terapêutica, Centro de Investigação, Instituto Português de Oncologia do Porto, 4200-072 Porto, Portugal

**Keywords:** pediatric leukemia, acute lymphoblastic leukemia, oral health, oral manifestations, dental care

## Abstract

Acute lymphocytic leukemia (ALL) is the most frequent form of all childhood leukemias, mostly affecting children between 2 and 4 years old. Oral symptoms, such as mouth ulcers, mucositis, xerostomia, Herpes or Candidiasis, gingival enlargement and bleeding, petechiae, erythema, mucosal pallor and atrophic glossitis, are very common symptoms of ALL and can be early signs of the disease. Secondary and tertiary complications, a direct effect of chemo and radiotherapy, are associated with more severe bleeding, higher susceptibility to infections, ulcerations, inflammation of the mucous membranes, osteoradionecrosis, xerostomia, taste alterations, trismus, carious lesions and dental abnormalities. Immunotherapy, though less toxic, causes oral dysesthesia and pain. Overall, the effects in the oral cavity are transient but there are long-term consequences like caries, periodontal disease and tooth loss that impair endodontic and orthodontic treatments. Also, dental abnormalities resulting from disturbed odontogenesis are known to affect a child’s quality of life. The medical dentist should identify these complications and perform appropriate oral care in tandem with other health professionals. Thus, poor oral hygiene can lead to systemic ALL complications. The aim of this review is to describe the oral complications in children with ALL who are undergoing chemo, radio or immunotherapy.

## 1. Introduction

Leukemia is the most common pediatric cancer in children under 15 years of age, accounting for 30% of all cancers [1]. The survival rate for a child at 3 years is 85% and, at 5 years, 81% [2]. Leukemia is clinically divided into acute and chronic leukemia, and the acute form can be fatal within days. On the basis of histogenicity, it is classified as lymphocytic or myelocytic [3].

According to the Global Cancer Observatory, in 2020, Portugal had an estimated number of 7.2% new cases of childhood leukemia and this type of cancer accounted for 44.5% of all other types of cancers [4].

In acute lymphocytic leukemia (ALL) there is a massive proliferation of lymphoblasts and the number of circulating mature cells decreases, leading to bone marrow failure syndrome [5,6]. It can affect the B cell lineage, which is the most frequent form of all childhood leukemias with a prevalence of 80%, whereas ALL affecting T cells is rare [7,8,9]. ALL mostly affects children between the age of 2 and 4 years old with overall survival (OS) above 90%, while at earlier ages this rate was lower than 10% [1,8]. The involvement of the Central Nervous System (CNS) in this disease is the major cause of mortality in children, accounting for one-third of the relapsing cases and also impairing the therapeutic approach [10,11,12]. It is underdiagnosed and, in most cases, asymptomatic. Neurological symptoms, such as loss of balance, headache, fainting, nausea or, more rarely, swallowing difficulties, are observed depending on the leukemic infiltration and the number of CNS areas involved [13,14].

ALL etiology combines genetic alterations and environmental factors, like alcohol, cigarette and drugs, which, during pregnancy, can cause genetic mutations of the fetus. However, radiation exposure, chemicals and infections can lead to the development of ALL. Thus, there are several subgroups of ALL according to polymorphisms or other genetic abnormalities like hyperdiploidy and hypodiploidy [1,15]. To diagnose ALL, a complete blood count, a bone marrow puncture and a biopsy are performed, and disease confirmation occurs when 25% or more of the cells in the bone marrow are lymphoblasts [9]. Using cytogenetic techniques or fluorescence in situ hybridization (FISH), specific chromosomal abnormalities can be detected, allowing for the identification of specific subgroups associated with different disease outcomes [16]. In ALL, the low number of red blood cells (anemia) causes fatigue, weakness, shortness of breath during normal physical activities, light-headedness, dizziness or faintness, headaches, and a pale complexion. Leukopenia causes recurrent fevers and frequent infections. The low platelet count is associated with easy bruising; red spots on the skin, called petechiae; frequent or severe nosebleeds; bleeding gums; and, in women, more frequent menstrual periods. Further symptoms that can occur include weight loss or loss of appetite, swollen glands, bone and joint pain, difficulty breathing and an enlarged spleen or liver [17,18,19,20].

The primary ALL treatment is chemotherapy, followed by radiotherapy and a bone marrow transplant. The therapeutic scheme usually lasts 2 years, until the child achieves total remission. In case of relapsing, after a bone marrow transplant, there are several clinical trials with new targeted precision therapies that can be used [21]. Nowadays, the use of radiotherapy is under re-evaluation due to its long-term effects. Target systemic therapies are now combined with low-intensity radiotherapy only in specific patients. In addition, the radiotherapy technique is becoming more precise due to image guidance allowing a reduction in toxicity, while still controlling the disease [22,23]. In recent years, immunotherapy has evolved and the OS of these children has increased, although there are still several side effects of the disease/treatments [17].

Oral symptoms are very common in ALL and there can be early signs of the disease, including mouth ulcers, mucositis, xerostomia, infections like Herpes or Candidiasis, gingival enlargement, and bleeding, petechiae, erythema, mucosal pallor and atrophic glossitis. The medical dentist should be aware of these symptoms during dental check-ups and, once they have been identified, react by prescribing additional tests or refer the patient to specialized professionals [3,6,24].

The aim of this narrative review is to describe the oral complications in children with ALL before, during and after chemotherapy, radiotherapy or immunotherapy. We intend to alert the medical dentist to the most common short and long-term effects of ALL in the oral cavity.

## 2. Oral Complications in Children with Acute Lymphoblastic Leukemia

Leukemia and its treatment can directly or indirectly affect the oral health of patients and can significantly reduce their quality of life. Different factors increase the risk of developing oral complications, including the patient’s age, gender, nutritional status, type of leukemia, neutrophil count before initiation of treatment, chemotherapy phase, oral health at baseline and oral hygiene [8,25].

Younger patients have a higher rate of post-treatment oral complications than adults. Moreover, the impact of treatments on developing dentition and orofacial growths is high. Oral manifestations and complications include repercussions of hemopathy and adverse effects of treatment. These can be classified as:

- Primary complications, which occur mainly due to the disease itself; these result from the infiltration of malignant cells into oral structures, such as the gum and bone, and gingival edema or tooth pain due to pulp infiltration.
- Secondary complications are usually associated with a direct effect of radiation therapy or chemotherapy, such as thrombocytopenia, anemia and granulocytopenia. These include a tendency to bleed or susceptibility to infections.
- Tertiary complications usually occur due to the complex interactions of therapy and its side effects, such as immunosuppression. They include ulcerations, inflammation of the mucous membranes, osteoradionecrosis, xerostomia, taste alterations, trismus or carious lesions [26].

Oral complications can have a rapid onset upon treatment initiation, with a higher frequency during the first week of chemotherapy. Adverse effects in the oral cavity are frequent, and their signs and severity are diverse [27].

### 2.1. Mucositis

One of the most common side effects occurring during chemotherapy is oral mucositis, also called stomatitis, which has a prevalence of around 40%. It is an inflammation of the mucous membranes, which initially presents as an erythema, often progressing to erosion with loss of the epithelial barrier, followed by ulceration. The most affected areas are the non-keratinized mucous membranes, namely the soft palate, oropharynx, oral and labial mucous membranes, the floor of the mouth, and the ventral and lateral surfaces of the tongue. The patient complains of burning, pain, tingling of the lips and a dry mouth. The pain associated with mucositis can cause difficulty eating, hydrating, and speaking, which can lead to weight loss, anorexia, cachexia and dehydration [18]. Recommended protocols include the use of mouthwash such as normal saline or sodium bicarbonate solutions. Salt and soda mouthwashes are also safe to use [28,29].

### 2.2. Xerostomia

The majority of childhood cancer survivors experience one or more late effects arising from childhood cancer treatment. Amongst these late effects, survivors may develop salivary gland dysfunction, such as hyposalivation (decreased salivary secretion) and/or xerostomia (oral dryness sensation due to reduced salivation [30]. Xerostomia is the second most common side effect of chemotherapy. Studies have shown that there is a direct relationship between radiation dose and a reduction in saliva [Jensen]. Salivary gland dysfunction is a significant and probably underestimated late effect and may negatively affect general health, as saliva maintains oral health by protecting the oral mucosa and teeth [31,32].

Sedatives, opiates, or antihistamines can also induce xerostomia, but their effects are minor compared to those of radiation therapy. Changes in saliva, both quantitative and qualitative, may appear shortly after induction of antineoplastic therapy. Radiation therapy causes fibrosis and degeneration of the salivary acinar cells, leading to necrosis of the main salivary glands. Saliva becomes more viscous and the proportion of organic matter increases. As a result, its color can change from transparent to opaque, white, or yellow. Its buffering capacity and its pH decrease, which induces a significant increase in the prevalence of infections such as candidiasis, periodontal diseases and caries [31]. The decreased flow and increased viscosity of saliva can cause difficulty chewing, swallowing, or speaking. Lack of saliva also hinders the functioning of the taste buds, causing taste impairment [33,34]. The child should rinse their mouth with sterile water, cold saline or sodium bicarbonate solution as often as possible to keep the tissues of the mouth clean and moist. This helps ensure the removal of thick saliva and food debris and reduces the risk of opportunistic infections. Artificial saliva based on carboxymethylcellulose can also be used, or salivary secretion can be stimulated via simple dietary measures such as eating raw vegetables, chewing sugar-free gums containing xylitol or by using pilocarpine (a cholinergic parasympathomimetic agent used to stimulate salivary flow) [35,36].

### 2.3. Dysgeusia

The presence of mucositis, as well as treatments, cause dysgeusia (altered taste) which can lead to difficult or even unsuitable nutrition. Some types of chemotherapy drugs can create a bad taste sensation, called the “venous taste phenomenon” due to diffusion of the drug into the oral cavity. Saliva becomes very viscous and unabundant and no longer allows the food bolus to reach the taste buds located in the posterior part of the tongue, which can also cause an alteration of taste. This loss or alteration of taste is often transient because the affected buds regenerate between 2 and 12 months. An alteration in taste can lead to a loss of appetite because children no longer like the taste of certain foods [37].

Management of this symptom requires dietary counseling and parents and the child must be alerted to the importance of good oral hygiene before the treatments [38].

### 2.4. Gingivitis and Gingival Bleeding

Children with ALL have around 90% prevalence of gingivitis, one of the most common oral manifestations of leukemia. Their risk of developing gingival inflammation is increased compared to that of healthy children [39].

In pediatric ALL, the frequency of gingivitis is increased not only during, but also after treatment, and can evolve, if poorly controlled, to periodontal disease [40].

In addition, gingivitis can manifest from simple bleeding from an inflamed gum to a bruise, hematoma or hemorrhage. Bleeding occurs due to thrombocytopenia (a decrease in platelet count), a consequence of the chemotherapeutic agents, and is aggravated by poor oral hygiene. Prevention is the most effective technique to avoid bleeding: this includes elimination of areas of potential traumatic areas (sharp restorations, fractured teeth) and pre-existing intraoral diseases before chemotherapy. Minor oral bleeding can be controlled with simple pressure using a compress soaked in a hemostatic solution, but major bleeding may require platelet transfusions. Some procedures, such as tooth extractions or periodontal surgery, must be performed when the child has a regular platelet count [20].

### 2.5. Caries

In children with ALL, an increase in dental caries is usually observed. However, dental caries does not occur because of the disease, or because of radiation therapy or chemotherapy. It is due to alterations in the functioning of the salivary glands, the tendency to have a soft and sweet diet, modification of the oral flora and the inability to maintain oral hygiene due to pain and gingival inflammation [41]. Post-radiation salivary gland damage reduces salivary secretion, makes saliva more acidic, and promotes highly cariogenic oral microflora such as *Streptococcus mutans* and *Lactobacillus*. Impaired dental health due to caries affects cosmesis, functioning and quality of life [42]. The role of the healthcare team is to encourage patients to maintain oral hygiene and inform them of the restrictions on sticky and sweet foods. Many pediatric medicines contain sugar to improve the taste and make them easier to take. It is advisable to avoid these drugs during periods of sleep and to drink water after ingestion [43,44].

### 2.6. Trismus

Trismus is defined as restricted opening of the mouth and it can be due to dental abscesses, trauma, local anesthesia to the mandible, head and neck radiation therapy, and chemotherapy. Thus, trismus associated with icteric appearance, general fatigue loss of appetite and swollen lymph nodes can point to the suspicion of ALL before a diagnosis [45]. The chemotherapeutic agents can cause edema and cell destruction while the radiotherapy-induced fibrotic can cause changes of the masticatory muscles—muscle fibrosis. The limited opening of the mouth can make correct oral hygiene difficult, disrupt eating and swallowing and further hamper the health of the oral cavity [46]. The child should complete regular exercises to stimulate mouth opening and closing and symptoms can be relieved with the administration of anti-inflammatory drugs and muscle relaxants [42].

### 2.7. Candida and Other Infections

Candidiasis is one of the most common opportunistic infections seen in children with leukemia and is caused primarily by *Candida albicans.* A dry oral environment favors the appearance of *Candida* species that stagnate on the oral soft tissues. The clinical signs are small adherent white spots, commonly called thrush, on the oral mucosa, tongue and palate. These small spots have the appearance of “curdled milk” which, when scraped, can be eliminated, and reveal small superficial wounds with slight bleeding. Moreover, there may be erythematous eroded areas, whose appearance is not only due to oral mucosa atrophy, but also due to increased vascularization. The child can also present angular cheilitis, forming a fissure at the labial commissure level. Due to immunosuppression caused by the disease or by the chemotherapeutic agents, various opportunistic infections, other than *Candida*, may occur [26]. The most observed viral infections are both caused by the *Herpesviridae* family: *Herpes simplex* and *Varicella-zoster*. *Herpes simplex* clinically manifests in the form of ulcers located at the corners of the mouth, lips, palate and gums. *Varicella zoster* is seen as multiple blisters, which show a protracted course. It may involve the lungs, central nervous system and liver and is associated with high morbidity. Its management will be almost exclusively palliative. Cytomegalovirus, Adenovirus and Epstein–Barr virus can also appear [39]. In all cases, various topical and systemic antifungal agents can be used: nystatin, clotrimazole and ketoconazole in the first case or fluconazole, itraconazole or ketoconazole in the second situation [47].

### 2.8. Osteoradionecrosis

Osteoradionecrosis is considered to be one of the most serious oral complications of radiation therapy. Radiation reduces the potential for tissue vascularization by damaging the endothelial lining of the vessels. Blood flow is reduced, thus limiting nutrient supply and defense cells. This leads to hypoxic conditions, resulting in vasculitis and ischemic necrosis of the bone—the most affected tissue, especially if it is subjected to a trauma, such as tooth extraction [48]. The most common site for osteonecrosis after head and neck radiation is the maxilla due to its poor vascularization and presence of teeth. Introduction of preventive oral hygiene and thorough dental assessment before and after radiation may result in a decreased incidence of osteoradionecrosis [49].

### 2.9. Oral Dysesthesia

Over the last decade, the prognosis for children with ALL has improved due to the use of precision medicine, identification of distinct subgroups and development of targeted therapies [50]. Immunotherapy, alone or combined with chemotherapy, is used for pediatric refractory/relapsing ALL (R/R ALL) and for Philadelphia-positive (Ph+) children, a rare and poor prognosis subgroup with a defective chromosome—22—resulting from a genetic translocation [51,52]. Tyrosine kinase inhibitors (TKI), monoclonal antibodies and chimeric antigen receptor T (CAR-T) cells are already used, with limited oral manifestations. TKI are associated with oral dysesthesia, specifically oral mucosal sensitivity, a type of oral toxicity not observed in patients receiving conventional chemotherapy and radiotherapy: there is a mucosal discomfort without histologic alterations. The child should avoid irritating foods and drinks, use topical analgesics for associated pain and maintain good oral hygiene [53,54].

**Figure 1:**
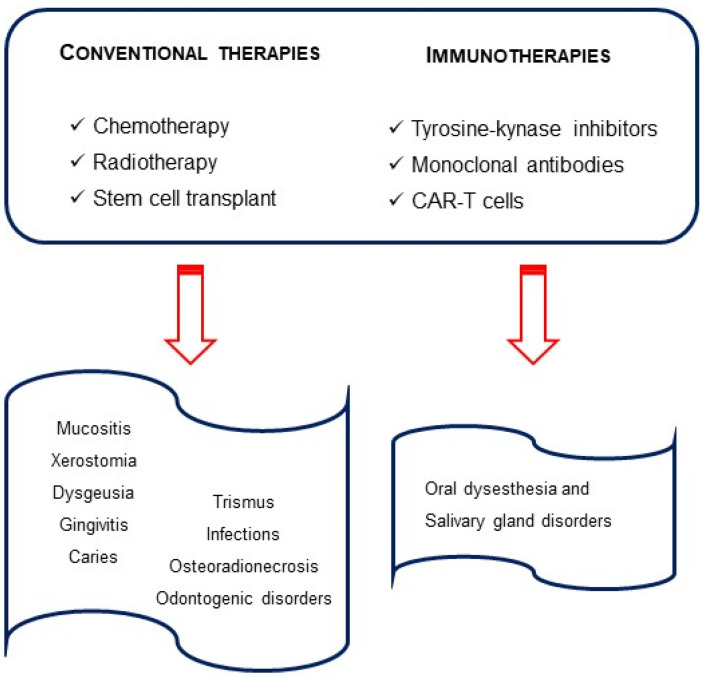
Therapies in pediatric acute lymphocytic leukemia and oral outcomes.

Blinatumomab, a monoclonal antibody, targeting CD19 antigen and anti-CD19 CAR-T cells are also approved for R/R ALL children with rare oral effects [55] They induce an immune response which can evolve to cytokine release syndrome (CRS) and neurological toxicity. The latter is associated with speaking and swallowing problems, difficulty with facial movements and xerostomia. Thus, these are all transient conditions [56,57]. The Food and Drug Administration (FDA)-approved therapies for children with ALL can be found in Figure 1.

Children with ALL frequently experience treatment-related pain. Some of the complications mentioned before, like mucositis and xerostomia, can also lead to constant jaw pain and dental sensitivity. This is often underestimated and undertreated in children, so early diagnosis will contribute to better decisions of the therapeutic strategy to be applied [44].

### 2.10. Dental Developmental Disorders

Dental developmental disorders are prevalent among children with ALL. Diagnosis before the age of 3 and dose-dependent alkylating agent therapy are the main risk factors for dental alterations [30]. Thus, there is strong evidence that dental abnormalities are more prevalent in children who have received chemotherapy compared to healthy children.

Disturbed odontogenesis has been demonstrated after the administration of numerous chemotherapeutic agents by interfering with the mitotic cycle of cancer cells [58]. The abnormalities are caused by the type, intensity, frequency of the treatment and age of the patients at ALL diagnosis. Thus, it has important consequences for the children’s dental development. The effects of chemo and radiotherapy on the orofacial area depend on the age of the patient at the beginning of treatment and on the radiation dose [59,60].

Anti-neoplastic therapies can lead to enamel dysplasias (discoloration and hypoplasia), as well as to radicular anomalies such as resorbed or tapered roots; delayed root formation; early apical closure; dental anomalies including V-shaped roots, delayed dental development or dental impaction; qne dental shape anomalies (microdontia, macrodontia, taurodontia), and dental numbers anomalies can also be found (hypodontia, agenesia, supernumerary teeth) [30,32,59,60,61]. Furthermore, after ALL, children are at a higher risk of developing dental caries, lesions of the enamel, discoloration of teeth or even early tooth loss, thus requiring the use of dental prosthesis [62].

This wide range of variations in dental structures is recognized as a side effect of childhood cancer therapy in long-term survivors of pediatric malignancies and may affect their quality of life [63].

**Figure 2:**
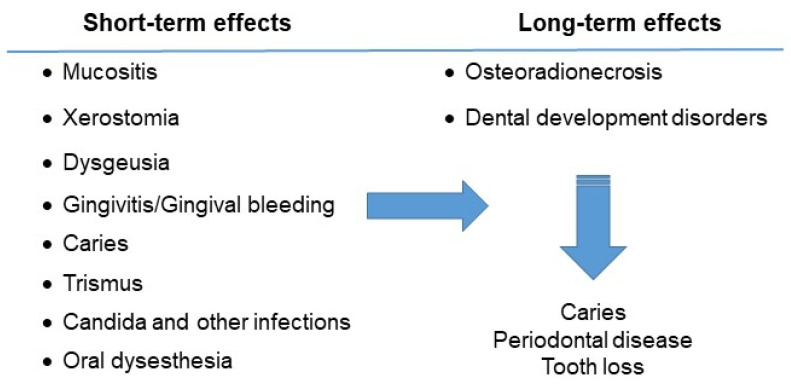
Oral complications of the child with acute lymphocytic leukemia.

The oral effects of the disease and drugs used for the treatment can be divided according to their duration. Most of the complications are short term and last only until the end of the therapeutic treatment. Others are considered long-term adverse effects, especially when the child’s odontogenesis is affected (Figure 2).

## 3. Dental Care and Management of the Child Patient

Children with ALL have unique oral health needs and are at risk of developing multiple associated oral and systemic complications. The American Academy of Pediatric Dentistry (AAPD) recognizes that the pediatric dental professional plays an important role in the diagnosis, prevention, stabilization and treatment of oral and dental problems that can compromise a child’s quality of life before, during and after cancer treatment. A multidisciplinary approach involving physicians, nurses, dentists, social workers, dieticians, and other related health professionals is essential to care for the child before, during and after immunosuppressive therapy and/or head and neck radiation [29].

Dental intervention must be prompt and efficient, considering the patient’s medical history, treatment protocol and health status. Oral complications are common and can disrupt the chemotherapy protocol, forcing healthcare providers to reduce the dose of treatments or even stop them. The medical dentist’s objectives in the oral care of the child with ALL are dependent on the treatment phase—before, during or after therapy. A dental evaluation is therefore necessary before starting any treatment to prevent or limit complications. During chemotherapy, the symptoms are more severe, affecting the mouth and resulting in pathologies ranging from spontaneous bleeding and infections to odontogenic disorders [64].

Contemporary recommendations for the management of dental and oral care of the child with ALL before, during or after chemotherapy, head and neck radiation or immunotherapy are discussed below. These are based upon the expert and/or consensus opinion of experienced researchers and clinicians [29].

### 3.1. Before Chemotherapy

The aims of chemotherapy and/or radiotherapy are:

(a) To identify and stabilize or eliminate possible sources of infection and local irritants in the oral cavity in order to avoid delaying cancer treatment or inducing other complications. Any existing lesions that might normally lie dormant can flare up and become life threatening once the child is immunosuppressed.
(b) To educate patients and parents about the importance of optimal oral care to minimize oral problems and discomfort before, during and after cancer treatment.
(c) To advise patients/parents about the possible short- and long-term side effects of cancer treatment in the oral cavity and craniofacial complex [65,66].

The pediatric objectives in the oral care of children with ALL are to reduce pain and discomfort; identify, stabilize or eliminate potential sources of infection; restore carious teeth; and improve oral hygiene. An interdisciplinary approach between dental surgeon and hematologist is crucial before implementing any dental treatment. Appropriate dental care before starting treatments should not be neglected, as oral infections can interfere with oncological treatment. Trauma associated with oral function and mucosal damage increases the risk of bleeding. Thus, dental consultation in a child newly diagnosed with the disease must be conducted immediately. There must be sufficient time to complete dental work before starting ALL treatment, as any existing lesions that might normally lie dormant can burst and become fatal once the child is immunocompromised [67]. Meticulous oral care is important to reduce the incidence and severity of oral sequelae of the treatment protocol. Both the parents and child should be educated about the importance of good oral hygiene practices throughout the entire oncology treatment, regardless of the child’s hematological status [66]. The goal is to improve the health and integrity of the mucous membranes. The use of a soft toothbrush followed by a mouthwash with chlorhexidine or bicarbonate solutions will be recommended. If there is spontaneous bleeding from the gums, brushing can be replaced by swabbing the gums using compresses soaked in antiseptic solution [44].

A clinical and radiographic evaluation is necessary before starting dental treatment in order to establish a treatment plan in coordination with the hematologist: scaling should be performed to reduce the inflammatory state of the soft tissues; any carious lesions should be treated with temporary or permanent restorations before starting chemotherapy. Pulp treatments are only necessary when clinical signs are evident before starting chemotherapy. Teeth with extensive lesions in the pulp, periapical lesions, as well as teeth with incomplete root canal fillings should be extracted to minimize the risk of systemic oral complications. Ideally, all dental extractions should be performed 3 weeks before starting cancer treatment to allow epithelization of the extraction site and adequate healing [17,18,24].

### 3.2. During Chemotherapy

The objectives of dental/oral care during cancer therapy are stated below:

(a) To maintain optimal oral health during cancer therapy.
(b) To manage any oral side effects that may develop because of the cancer therapy.
(c) To reinforce the patient and parents’ education regarding the importance of optimal oral care to minimize oral problems/discomfort during treatment [7,66].

The child may find oral lesions so painful that brushing their teeth becomes almost impossible. In this case tooth brushing should be replaced by a swab using compresses soaked in bicarbonate or chlorhexidine-based solution combined with an antifungal and an anesthetic. Dental care can be resumed, between two cycles of chemotherapy, after checking the blood count, and care must be limited to primary emergencies. Every treatment must be carried out after consultation with the pediatric oncologist and hematologist [7]. However, during invasive procedures, blood tests may be necessary. Dental sequelae may appear in children who have undergone chemotherapy or radiation therapy during the period of odontogenesis, including dental agenesis, coronary abnormality (hypoplasia, microdontia) and root abnormality. These anomalies do not require any treatment; in this case, simple monitoring until dental maturation is necessary [7].

Children who have received a bone marrow transplant will not be able to have dental procedures for a year due to profoundly impaired immune function. In addition, there is a high incidence after the bone marrow transplant of Graft versus Host Disease, a type of rejection that has systemic and oral effects. The latter can include rampant caries and squamous cell carcinoma [19].

During treatment, the medical dentist should provide adequate and appropriate oral care to prevent and minimize oral complications and improve the patient’s quality of life. Involving the parents in the child’s oral health should ensure the success of the treatment [19,66].

### 3.3. After Chemotherapy

The objectives of a dental/oral examination after conclusion of cancer therapy are:

(a) To maintain optimal oral health.
(b) To reinforce the importance of optimal oral and dental care for life to the patient/parents [66].

Children in complete remission can be treated without precaution for conservative care [66]. Educating parents and health care providers in early prevention is vital to minimize the harmful effects of cancer treatment. Comprehensive clinicians and dentists with expertise in survivor cancer care are not accessible for all survivors. However, a reduction in the burden of dental complications among vulnerable survivors is possible with early and regular dental follow-up [42].

The medical dentist plays a major role in monitoring disease relapse and the development of new blood diseases and solid tumors. After treatment, the follow-up should be short—with 3 to 6 months between each appointment—and the medical dentist should manage any manifestations and advice about good oral hygiene practice. After 2 years, the appointments can be conducted at more spaced-out intervals [56].

Orthodontic treatment can begin or resume after completion of all antineoplastic treatments, at least after 2 years of remission, when the risk of relapse is reduced, and the patient is no longer taking immunosuppressive drugs [56]. Thus, the risk of developing complications is always increased due to the disease and its treatments, especially if there are alterations in bone metabolism and oral mucosa sensitivity that can cause ulcerations [67,68]. The treatment should be of short duration and the orthodontic materials should be nickel free. Titanium should be used, due to its corrosion resistance and minimal release of particles into the oral cavity [69]. Furthermore, root resorption, which is common in orthodontic therapy, should be particularly considered, as it is one of the development abnormalities commonly associated with ALL. Root resorption risk should be minimized, applying low forces and short treatments with periodic periapical radiographs [70].

Besides orthodontic treatment, prosthetic and endodontic procedures, and esthetic restorations of the enamel defects are often necessary [18].

Successful management of oral complications, including systemic infections of oral origin, should begin with the oral examination, the introduction of comprehensive oral hygiene measures and definitive dental interventions prior to initiation of cancer therapy. The incorporation of a pediatric dentist in the multidisciplinary team in childhood cancer, and the establishment of standardized protocols based on prevention, and not just the treatment of the complications is, accordingly, necessary in order to ensure successful cancer treatment and the best quality of life [65].

## 4. Concluding Remarks

Oral lesions in children with leukemia, the most common pediatric hematologic cancer, can occur due to the disease or the treatment. Over the last decade, pediatric chemotherapy protocols have evolved, although oral health is always compromised, both by transient and permanent conditions, such as a high risk of caries, microbiota dysbiosis and dental abnormalities. It is of outmost importance to develop new personalized strategies and therapeutic options without impairing the oral health. The use of FDA-approved TKI, CAR-T cells and monoclonal antibodies have rare adverse oral effects, ultimately improving the child’s quality of life. Thus, the medical dentist plays a major role in the early diagnosis and improved quality of life of children with ALL. A multidisciplinary team of pediatric dentistry and oncologists could develop a standardized protocol to implement during and after cancer therapy, thus ensuring the best outcome for the pediatric patient. In addition, dental professionals must be prepared to meet the unique needs of long-term cancer survivors because of the increasing survival rates of childhood cancers.

## Data Availability

Not applicable.

## References

[1] Madhusoodhan P.P., Carroll W.L., Bhatla T. (2016). Progress and Prospects in Pediatric Leukemia. Curr. Probl. Pediatr. Adolesc. Health Care.

[2] Fathi A., Mirzarahimi M., Farajkhah H. (2021). Réponse à un schéma chimiothérapeutique administré à des enfants atteints de LAL à cellules pré-B à risque élevé selon le protocole COG. Can. Oncol. Nurs. J..

[3] Lim H.-C., Kim C.-S. (2014). Oral signs of acute leukemia for early detection. J. Periodontal Implant. Sci..

[4] (2022). Global Cancer Observatory (iarc.fr). https://gco.iarc.fr/today/home.

[5] Seth R., Singh A. (2015). Leukemias in Children. Indian J. Pediatr..

[6] Tarlock K., Dahl G., Lacayo N. (2018). Acute myeloid leukemia in children. Wintrobe’s Clinical Hematology.

[7] Zimmermann C., Meurer M.I., Grando L.J., Del Moral J.G., Rath I.B.d.S., Tavares S.S. (2015). Dental Treatment in Patients with Leukemia. J. Oncol..

[8] Babu K.L.G., Mathew J., Doddamani G.M., Narasimhaiah J.K., Naik L.R.K. (2016). Oral health of children with acute lymphoblastic leukemia: A review. J. Orofac. Sci..

[9] Svsg N. (2016). Dental Care of Children with Leukemia—An Overview. J. Pediatr. Neonatal Care.

[10] Imbach P., Kuhne T., Arceci R. (2011). Pediatric Oncology Book 2011.

[11] Baytan B., Evim M.S., Güler S., Güneş A.M., Okan M. (2015). Acute Central Nervous System Complications in Pediatric Acute Lymphoblastic Leukemia. Pediatr. Neurol..

[12] Deak D., Gorcea-Andronic N., Sas V., Teodorescu P., Constantinescu C., Iluta S., Pasca S., Hotea I., Turcas C., Moisoiu V. (2021). A narrative review of central nervous system involvement in acute leukemias. Ann. Transl. Med..

[13] Del Principe M.I., Maurillo L., Buccisano F., Sconocchia G., Cefalo M., De Santis G., Di Veroli A., Ditto C., Nasso D., Postorino M. (2014). Central nervous system involvement in adult acute lymphoblastic leukemia: Diagnostic tools, prophylaxis, and therapy. Mediterr. J. Hematol. Infect. Dis..

[14] Johnston D.L., Alonzo T.A., Gerbing R.B., Aplenc R., Woods W.G., Meshinchi S., Gamis A.S. (2017). Central nervous system disease in pediatric acute myeloid leukemia: A report from the Children’s Oncology Group. Pediatr. Blood Cancer.

[15] Tebbi C.K. (2021). Etiology of Acute Leukemia: A Review. Cancers.

[16] Van Delft F.W., Saha V., Goulden N.J., Steward C.G. (2004). Molecular Techniques to Improve Outcome in Childhood ALL pp111-12. Pediatric Hematology Methods and Protocols.

[17] Cho S.Y., Cheng A.C., Cheng M.C. (2018). Oral care for children with leukaemia. Hong Kong Med. J..

[18] Valéra M.-C., Noirrit-Esclassan E., Pasquet M., Vaysse F. (2015). Oral complications and dental care in children with acute lymphoblastic leukaemia. J. Oral Pathol. Med..

[19] Lowal K., Alaizari N., Tarakji B., Petro W., Hussain K., Altamimi M. (2015). Dental Considerations for Leukemic Pediatric Patients: An Updated Review for General Dental Practitioner. Mater. Socio Medica.

[20] Francisconi C.F., Caldas R.J., Martins L.J.O., Rubira C.M.F., Santos P.S.d.S. (2016). Leukemic Oral Manifestations and their Management. Asian Pac. J. Cancer Prev..

[21] Rafei H., Daher M., Rezvani K. (2020). Chimeric antigen receptor (CAR) natural killer (NK)-cell therapy: Leveraging the power of innate immunity. Br. J. Haematol..

[22] Packer R.J., Pfister S., Bouffet E., Avery R., Bandopadhayay P., Bornhorst M., Bowers D.C., Ellison D., Fangusaro J., Foreman N. (2016). Pediatric low-grade gliomas: Implications of the biologic era. Neuro-Oncology.

[23] Shen C.J., Terezakis S.A. (2021). The Evolving Role of Radiotherapy for Pediatric Cancers with Advancements in Molecular Tumor Characterization and Targeted Therapies. Front. Oncol..

[24] Ritwik P., Chrisentery-Singleton T.E. (2020). Oral and dental considerations in pediatric cancers. Cancer Metastasis Rev..

[25] Mathur V.P., Kalra G., Dhillon J.K. (2012). Oral health in children with leukemia. Indian J. Palliat. Care.

[26] Wang Y., Zeng X., Yang X., Que J., Du Q., Zhang Q., Zou J. (2021). Oral Health, Caries Risk Profiles, and Oral Microbiome of Pediatric Patients with Leukemia Submitted to Chemotherapy. BioMed Res. Int..

[27] Lula E.C.d.O., Lula C.E.d.O., Alves C.M.C., Lopes F.F., Pereira A.L.A. (2007). Chemotherapy-induced oral complications in leukemic patients. Int. J. Pediatr. Otorhinolaryngol..

[28] Brown T.J., Gupta A. (2015). Management of Cancer Therapy–Associated Oral Mucositis. J. Oncol. Pract..

[29] American Academy of Pediatric Dentistry (AAPD) (2022). Dental Management of Pediatric Patients Receiving Immunosuppressive Therapy and/or Head and Neck Radiation.

[30] Stolze J., Vlaanderen K.C.E., Holtbach F.C.E.D., Teepen J.C., Kremer L.C.M., Loonen J.J., Broeder E.v.D.-D., Heuvel-Eibrink M.M.v.D., van der Pal H.J.H., Versluys B. (2021). Long-Term Effects of Childhood Cancer Treatment on Dentition and Oral Health: A Dentist Survey Study from the DCCSS LATER 2 Study. Cancers.

[31] Jensen S., Pedersen A., Reibel J., Nauntofte B. (2003). Xerostomia and hypofunction of the salivary glands in cancer therapy. Support. Care Cancer.

[32] Stolze J., Teepen J.C., Raber-Durlacher J.E., Loonen J.J., Kok J.L., Tissing W.J.E., de Vries A.C.H., Neggers S.J.C.M.M., Broeder E.v.D.-D., Heuvel-Eibrink M.M.v.D. (2022). Prevalence and Risk Factors for Hyposalivation and Xerostomia in Childhood Cancer Survivors Following Different Treatment Modalities—A Dutch Childhood Cancer Survivor Study Late Effects 2 Clinical Study (DCCSS LATER 2). Cancers.

[33] Jensen S.B., Vissink A., Limesand K.H., E Reyland M. (2019). Salivary Gland Hypofunction and Xerostomia in Head and Neck Radiation Patients. J. Natl. Cancer Inst.–Monogr..

[34] Silva I.M., Donaduzzi L.C., Perini C.C., Couto S.A., Werneck R.I., de Araújo M.R., Kurahashi M., Johann A.C., Azevedo-Alanis L.R., Vieira A.R. (2021). Association of xerostomia and taste alterations of patients receiving antineoplastic chemotherapy: A cause for nutritional concern. Clin. Nutr. ESPEN.

[35] Villa A., Connell C.L., Abati S. (2014). Diagnosis and management of xerostomia and hyposalivation. Ther. Clin. Risk Manag..

[36] Johnson J.T., Ferretti G.A., Nethery W.J., Valdez I.H., Fox P.C., Ng D., Muscoplat C.C., Gallagher S.C. (1993). Oral pilocarpine for post-irradiation xerostomia in patients with head and neck cancer. New Engl. J. Med..

[37] Brink M.v.D., Ijpma I., van Belkom B., Fiocco M., Havermans R.C., Tissing W.J.E. (2021). Smell and taste function in childhood cancer patients: A feasibility study. Support. Care Cancer.

[38] Munankarmi D. (2017). Management of Dysgeusia related to Cancer. J. Lumbini Med. Coll..

[39] Ponce-Torres E., Ruíz-Rodríguez M.d.S., Alejo-González F., Hernández-Sierra J.F., de Pozos-Guillén A. (2010). Oral Manifestations in Pediatric Patients Receiving Chemotherapy for Acute Lymphoblastic Leukemia. J. Clin. Pediatr. Dent..

[40] Kaskova L.F., Yanko N.V., Vashchenko I.Y. (2019). Gingival health in children in the different phases of acute lymphoblastic leukemia. Curr. Issues Pharm. Med. Sci..

[41] Shayani A., Aravena P.C., Rodríguez-Salinas C., Escobar-Silva P., Diocares-Monsálvez Y., Angulo-Gutiérrez C., Rivera C. (2021). Chemotherapy as a risk factor for caries and gingivitis in children with acute lymphoblastic leukemia: A retrospective cohort study. Int. J. Paediatr. Dent..

[42] Gawade P.L., Hudson M.M., Kaste S.C., Neglia J.P., Constine L.S., Robison L.L., Ness K.K. (2014). A systematic review of dental late effects in survivors of childhood cancer. Pediatr. Blood Cancer.

[43] Al Humaid J. (2018). Sweetener content and cariogenic potential of pediatric oral medications: A literature. Int. J. Health Sci..

[44] American Academy of Pediatric Dentistry (AAPD) (2013). Guideline on Dental Management of Pediatric Patients Receiving Chemotherapy, Hematopoietic Cell Transplantation, and/or Radiation Therapy.

[45] Katz J., Peretz B. (2002). Trismus in a 6 year old child: A manifestation of leukemia?. J. Clin. Pediatr. Dent..

[46] Satheeshkumar P., Mohan M.P., Jacob J. (2014). Restricted mouth opening and trismus in oral oncology. Oral Surg. Oral Med. Oral Pathol. Oral Radiol..

[47] Yigit M., Bilir A., Yüksek S.K., Kaçar D., Özbek N.Y., Yarali H.N. (2022). Antifungal Therapy in Pediatric Acute Lymphoblastic Leukemia. J. Pediatr. Hematol./Oncol..

[48] Brivio E., Cossio A., Borra D., Silvestri D., Prunotto G., Colombini A., Verna M., Rizzari C., Biondi A., Conter V. (2022). Osteonecrosis in paediatric acute lymphoblastic leukaemia: Incidence, risk factors, radiological patterns and evolution in a single-centre cohort. Br. J. Haematol..

[49] Kuhlen M., Kunstreich M., Gökbuget N. (2021). Osteonecrosis in Adults with Acute Lymphoblastic Leukemia: An Unmet Clinical Need. Hemasphere.

[50] Pui C.-H. (2020). Precision medicine in acute lymphoblastic leukemia. Front. Med..

[51] Lejman M., Kuśmierczuk K., Bednarz K., Ostapińska K., Zawitkowska J. (2021). Targeted Therapy in the Treatment of Pediatric Acute Lymphoblastic Leukemia—Therapy and Toxicity Mechanisms. Int. J. Mol. Sci..

[52] Ribera J.-M., Chiaretti S. (2022). Modern Management Options for Ph+ ALL. Cancers.

[53] Vigarios E., Epstein J.B., Sibaud V. (2017). Oral mucosal changes induced by anticancer targeted therapies and immune checkpoint inhibitors. Support. Care Cancer.

[54] Partanen M., Alberts N.M., Conklin H.M., Krull K.R., Pui C.-H., Anghelescu D.A., Jacola L.M. (2022). Neuropathic pain and neurocognitive functioning in children treated for acute lymphoblastic leukemia. Pain.

[55] Jasinski S., Reyes F.A.D.L., Yametti G.C., Pierro J., Raetz E., Carroll W.L. (2020). Immunotherapy in Pediatric B-Cell Acute Lymphoblastic Leukemia: Advances and Ongoing Challenges. Pediatr. Drugs.

[56] Lv M., Liu Y., Liu W., Xing Y., Zhang S. (2022). Immunotherapy for Pediatric Acute Lymphoblastic Leukemia: Recent Advances and Future Perspectives. Front. Immunol..

[57] Malczewska M., Kośmider K., Bednarz K., Ostapińska K., Lejman M., Zawitkowska J. (2022). Recent Advances in Treatment Options for Childhood Acute Lymphoblastic Leukemia. Cancers.

[58] Jodłowska A., Postek-Stefańska L. (2022). Systemic Anticancer Therapy Details and Dental Adverse Effects in Children. Int. J. Environ. Res. Public Health.

[59] Lupi S.M., Baena A.R.Y., Cervino G., Todaro C., Rizzo S. (2018). Long-Term Effects of Acute Myeloid Leukemia Treatment on the Oral System in a Pediatric Patient. Open Dent. J..

[60] Minicucci E.M., Lopes L.F., Crocci A.J. (2003). Dental abnormalities in children after chemotherapy treatment for acute lymphoid leukemia. Leuk. Res..

[61] Lauritano D., Petruzzi M. (2012). Decayed, missing and filled teeth index and dental anomalies in long-term survivors leukaemic children: A prospective controlled study. Med. Oral Patol. Oral Cir. Bucal..

[62] Proc P., Szczepańska J., Skiba A., Zubowska M., Fendler W., Młynarski W. (2016). Dental Anomalies as Late Adverse Effect among Young Children Treated for Cancer. Cancer Res. Treat..

[63] Çetiner D., Çetiner S., Uraz A., Alpaslan G.H., Alpaslan C., Memikoğlu T.U.T., Karadeniz C. (2019). Oral and dental alterations and growth disruption following chemotherapy in long-term survivors of childhood malignancies. Support. Care Cancer.

[64] Otmani N., Nachef M., Alaoui F.M. (2004). Prise en charge bucco-dentaire de l’enfant atteint de leucémie aiguë. Revue d’Odonto-Stomatologie.

[65] Ferrández-Pujante A., Pérez-Silva A., Serna-Muñoz C., Fuster-Soler J.L., Galera-Miñarro A.M., Cabello I., Ortiz-Ruiz A.J. (2022). Prevention and Treatment of Oral Complications in Hematologic Childhood Cancer Patients: An Update. Children.

[66] Padmini C., Bai K.Y. (2014). Oral and Dental Considerations in Pediatric Leukemic Patient. ISRN Hematol..

[67] Gholman R.R., El Meligy O.A., Felemban E.H. (2019). Dental Rehabilitation of a Child with Acute Lymphocytic Leukemia: A Case Report. Int. J. Clin. Pediatr. Dent..

[68] Hassan A., Manaf A., Zikra A., Burke M., Najla N. (2019). Oral and dental management of people with myelodysplastic syn-dromes and acute myeloid leukemia: A systematic search and evidence based clinical guidance. Spec Care Dent..

[69] Costa M.T., Lenza M.A., Gosch C.S., Costa I., Ribeiro-Dias F. (2007). In vitro evaluation of corrosion and cytotoxicity of or-thodontic brackets. J. Dent. Res..

[70] Krishnan V., Davidovitch Z. (2006). Cellular, molecular, and tissue-level reactions to orthodontic force. Am. J. Orthod. Dentofac. Orthop..

